# Muscular HSP70 content is higher in elderly compared to young, but is normalized after 12 weeks of strength training

**DOI:** 10.1007/s00421-021-04633-4

**Published:** 2021-03-07

**Authors:** K. T. Cumming, N. H. Kvamme, L. Schaad, I. Ugelstad, T. Raastad

**Affiliations:** 1 Department of Physical Performance, Norwegian School of Sport Sciences, Ullevål Stadion, P.O. 4014, 0806 Oslo, Norway; 2 Institute of Anatomy, University of Bern, Bern, Switzerland

**Keywords:** Heat shock protein, Strength training, Functional training, Aging, Skeletal muscle

## Abstract

**Purpose:**

Aging is associated with increased myocellular stress and loss of muscle mass and function. Heat shock proteins (HSPs) are upregulated during periods of stress as part of the cells protective system. Exercise can affect both acute HSP regulation and when repeated regularly counteract unhealthy age-related changes in the muscle. Few studies have investigated effects of exercise on HSP content in elderly. The aim of the study was to compare muscular HSP levels in young and elderly and to investigate how training affects HSP content in muscles from aged males and females.

**Methods:**

Thirty-eight elderly were randomized to 12 weeks of strength training (STG), functional strength training (FTG) or a control group (C). To compare elderly to young, 13 untrained young performed 11 weeks of strength training (Y). Muscle biopsies were collected before and after the intervention and analyzed for HSP27, αB-crystallin and HSP70.

**Results:**

Baseline HSP70 were 35% higher in elderly than in young, whereas there were no differences between young and elderly in HSP27 or αB-crystallin. After the training intervention, HSP70 were reduced in STG (− 33 ± 32%; *P* = 0.001) and FTG (− 28 ± 30%; *P* = 0.012). The decrease in HSP70 was more pronounced in the oldest. In contrast, Y increased HSP27 (134 ± 1%; *P* < 0.001) and αB-crystallin (84 ± 94%; *P* = 0.008).

**Conclusion:**

Twelve weeks of STG or FTG decreased the initial high levels of HSP70 in aged muscles. Thus, regular strength training can normalize some of the increases in cellular stress associated with normal aging, and lead to a healthier cellular environment in aged muscle cells.

**Supplementary Information:**

The online version contains supplementary material available at 10.1007/s00421-021-04633-4.

## Introduction

Aging is associated with declines in muscle mass and function, termed sarcopenia. It is well documented that strength training can counteract the decline in muscle mass and improve mobility and quality of life in the elderly (Narici and Maffulli 2010). In addition to the well-documented morphological changes observed during aging, changes at the cellular and biochemical level with negative effects on muscle quality also occurs (Aas et al. 2019). The group of heat shock proteins (HSPs) is a part of the cellular defense against a variety of different stressors, and they are involved in protection of proteins and cell structures during exercise stress and in the recovery of muscle function after exercise (Morton et al. 2009). Their biological role in exercising muscles is related to the prevention of damage and the recognition and repair of damaged proteins (Paulsen et al. 2009). All these processes seem to be affected by normal aging.

In general, the expression of heat shock proteins seems to increase in aging cells (Tower 2009). The elevated expression of heat shock proteins has been linked to increased oxidative stress (Wallen et al. 1997; Adrie et al. 2000), low-grade inflammation (Njemini et al. 2011; Beyer et al. 2012), and reduced capacity in removal of damaged proteins and organelles (Kikis et al. 2010). Only a few studies have investigated and compared basal levels of HSPs in skeletal muscles from young and elderly and the results are conflicting. In aged rodents, lower basal levels of HSP70 has been reported, whereas the 22 kDa HSP αB-crystallin has been found to be higher (Vasilaki et al. 2002; Doran et al. 2007). In contrast, two studies did not find any differences in HSP70 between young and aged rats (Naito et al. 2001; Starnes et al. 2005), and one study reported elevated levels of both HSP70 and HSP27 in aged rats (Siu et al. 2004). In humans, limited number of studies exist, but the results seem to be more consistent. Cobley et al. (2014) and Thalacker-Mercer et al. (2010) found higher HSP70 content in skeletal muscles from elderly compared to young. Beltran Valls et al. (2015) reported no significant differences of neither αB-crystallin, HSP27 nor HSP70 between young, healthy elderly or sarcopenic elderly. The low number of participants in this study might have precluded significant differences between groups. Recently, we reported increased levels of HSP70 and αB-crystallin in pre-frail and frail elderly compared to young individuals, but the levels in healthy elderly were more comparable to the young (Aas et al. 2019). Similarly, Joseph et al. (2012) reported higher levels of HSP70 in low-functioning compared to high-functioning elderly individuals. Consequently, frailty or lack of muscular activity might be a more important factor for the observed changes in HSP levels in muscles than aging per se. Despite some conflicting results, there seems to be a trend toward higher basal levels of HSPs, especially HSP70, in aging human muscles, but more studies are needed to confirm this trend and to investigate whether exercise interventions can counteract indices of increased cellular stress with aging.

In general, high intensity exercise increases the HSPs content in skeletal muscles acutely (Liu et al. 2000, 2004; Paulsen et al. 2007). The acute HSP response is associated with the protection of intact proteins and subsequent controlled removal of damaged proteins and structures, recovery processes, and the improved tolerability against similar exercise stress (Locke and Tanguay 1996; Vasilaki et al. 2002). Interestingly, aged muscles seem to have a dimiished HSP response to exercise and heat stress compared to young (Vasilaki et al. 2002; Locke and Tanguay 1996; Kregel et al. 1995), and in some studies, this has been explained by higher basal levels of HSPs in the muscles of elderly (Nilsen et al. 2016). In theory, chronic exercise of aged muscles restoring muscle function and reducing cellular stress should have the potential to reduce basal HSP levels. In line with this, Beltran Valls et al. (2013) reported reduced HSP levels in old participants after 12 weeks of resistance exercise. In contrast, two animal studies reported increased HSP levels in response to endurance training and electrically evoked resistance training in aged muscles (Naito et al. 2001; Murlasits et al. 2006). The conflicting results may be a consequence of different responses between species, selective responses to different training modalities, or a result of the timing of muscle samples and exercise (e.g., acute responses from the last exercise session may still be present). Consequently, more studies are needed to investigate the effect of regular training on HSP levels in aged human muscles and whether different exercise modalities exert different responses.

To our knowledge, no studies involving humans have been conducted to investigate the response of training on HSP content in muscles of aged males and females. Furthermore, the response to training modality and intensity on the HSPs in the elderly is currently unknown. The aim of the present study was therefore to investigate the training response on HSP content in different cellular fractions from elderly and young participants. We also aimed to investigate how two different types of strength training modalities, with different loads and muscle actions, affected HSP content in elderly males and females.

## Methods

The experimental design, training programs and results on changes in lean mass, muscle strength and function for the elderly participants have previously been described in detail (Solberg et al. 2013) and for the young participants (Paulsen et al. 2011). The present study includes data from a subgroup of the study population from these studies volunteering for a muscle biopsy.

### Participants

Thirty-eight active but untrained elderly females (*N* = 28) and males (*N* = 10) and 13 active but untrained young males volunteered to give a muscle biopsy before and after the training intervention. Participant characteristics at baseline are presented in Table 1. All participants gave written informed consent before entering and volunteering to a muscle biopsy and were informed about potential risks related to all parts of the experiment and the muscle biopsy procedure. The study was approved by the Regional Ethics Committee of Southern Norway and was performed in accordance with the standards of the Helsinki Declaration.

**Table 1:** Participant characteristics at baseline

	STG (*n* = 16)	FTG (*n* = 11)	C (*n* = 11)	YSTG (*n* = 13)
Age (years)	76 ± 6	73 ± 5	76 ± 4	26 ± 5†
Height (cm)	165 ± 7	170 ± 7	166 ± 9	183 ± 9†
Body mass (kg)	75 ± 17	71 ± 13	71 ± 13	81 ± 13
BMI	27 ± 5	25 ± 4	26 ± 4	24 ± 3
1RM (kg)	47 ± 8	48 ± 14	40 ± 6	71 ± 16†
Sex	♀ 12, ♂ 4	♀ 7, ♂ 4	♀ 9, ♂ 2	♂13

*STG* strength training group; *FTG* functional training group; *C* control group; *YSTG* young strength training group; *BMI* body mass index; *1RM* one repetition maximum in knee extention

♀Number of females; ♂number of males; †sig. (*p* < 0.05) different from elderly

### Experimental design

The training intervention with the elderly participants was completed over 12 weeks and was conducted as a single-blinded, randomized controlled trial. Based on baseline physical testing, the participants were stratified and randomly assessed to one of the following groups: strength training (STG; *n* = 16), functional strength training (FTG; *n* = 11) or a non-strength training control group (C; *n* = 11). The non-strength training control group consisted of four non-training participants, and seven participants involved in light endurance-type activities such as hiking and aerobics. The participants involved in the endurance activities did not differ in any of the variables measured in the present study. All training was supervised by qualified fitness instructors and done three times per week and lasted about one hour including warm up. The training intervention with the young participants was completed over 11 weeks and was conducted as a single-blinded, randomized controlled trial. The training sessions for the young participants were supervised once per week or more if needed.

### Strength training group (STG)

A whole-body strength training program was completed during each session, with two exercises focusing on the knee extensors (squat and knee extension). Each exercise consisted of 1–3 sets of 4-12RM. The training load was increased over the training period with variation in exercise load in each session (mix of linear periodization and daily undulation periodization). Two of the weekly training sessions were done to failure separated by one session with submaximal loads. Details about the training program are available in supplemental table s1.

### Functional strength training group (FTG)

Each training session consisted of a circuit training with exercises to mimic daily activities like chair rise, case lifting, bench-stepping, and loaded stair climbing. During the first 6 weeks, each exercise session consisted of two rounds with loads corresponding to 80% of 15RM on the first and 15RM on the last round on the exercise circuit. After six weeks, during two of the training sessions (day 1 and 3), the exercise load was increased to two full rounds of loads corresponding to 12RM. Details about the training program are available in supplemental table s2.

### Elderly control group (C)

The non-training control group was asked not to do any training during the 12-week intervention period and to continue with their normal daily activities. The non-training control group was allowed to participate in one of the training groups in the subsequent trail cycle. The endurance training-control group performed three sessions of light endurance training per week. The exercise sessions consisted of aerobics, Nordic walking, and hiking. The aerobic classes consisted of simple rhythmic and coordinative exercises to music. Nordic walking was conducted as interval training with 4 × 2 min walking with 1-min rest the first 6 weeks and 8 × 1 min with 30 s rest the last 6 weeks. Hiking was done in rugged terrain.

### Young strength training group (YSTG)

The training with the young participants consisted of a whole-body strength training program, with three exercises for the leg muscles (one exercise for the knee flexors) and five exercises for the upper body. The training load varied between 7 and 10RM and was completed in 1–3 series. Two of the weekly training sessions were done to failure separated by one session with submaximal loads. Details about the training program are available in supplemental table s3.

An overview for comparison of the different training programs is presented in Table 2.

**Table 2:** Overview of the different training programs

	Training frequency per week	No. of exercises affecting m.vastus lateralis	Training intensity	No. of exercise sets affecting m.vastus lateralis	Repetitions lifted affecting m.vastus lateralis during the intervention
STG	3	2	4-12RM + 80% of 10RM	2–3	1448
FTG	3	3–4	12-15RM + 15 reps with 20RM loads	2	3072
YSTG	3	2	7-10RM	3	1530

*STG* strength training group; *FTG* functional training group; *YSTG* young strength training group; *RM* repetition maximum

### Strength test

One repetition maximum (1RM) was measured for the quadriceps muscles in leg extension as previously described in detail for the elderly (Solberg et al. 2013) and young participants (Rønnestad et al. 2007). Briefly, after a standardized warm up of three sets with gradually increasing loads, the participants increased loads until failure to perform the required movement. The highest load lifted during the test was defined as 1RM. The rest between sets was approximately 3–4 min. The testing procedure was standardized and performed identically before and after the training intervention.

### Muscle sampling and pre-analytic handling

Muscle biopsies were sampled from the mid-portion of *m. vastus lateralis* before and 2–5 days after the last exercise session (week 11/12). The insertion of repeated biopsies was placed 3 cm proximally from the previous biopsy site. Under local anesthesia (Xylocain adrenalin, 10 mg/ml + 5 µg/ml, AstraZeneca PLC, London, UK), approximately 200 mg (2–3 × 50–150 mg) of muscle tissue was obtained with a modified Bergström-technique. Biopsy samples used for protein analyses were gently removed from the biopsy needle using a sterile syringe and quickly washed in physiological saline. Fat, connective tissue, and blood were discarded before quickly frozen in 2-methyl butane down on dry ice. All muscle samples were stored at – 80 °C until homogenization. For extraction of proteins, about 50 mg of muscle tissue were homogenized and fractionated into a cytosol-, membrane-, nuclear- and cytoskeletal fractions using a commercial fractionation kit (ProteoExtract Subcellular Proteo Extraction Kit, 539790, Calbiochem, EMD Biosciences, Schwalbach, Germany) in accordance to the manufacturer’s procedures. Homogenized samples were then aliquoted and re-frozen and stored at − 80 °C until further analyses. Protein concentrations were assessed in triplicates with a commercial kit (BioRad DC protein microplate assay, 0113, 0114, 0115, Bio-Rad, Hercules, CA, USA), a filter photometer (Expert 96, ASYS Hitech, UK) and calculated from the provided software (Kim, ver. 5.45.0.1, Daniel Kittrich, Prague, Czech Republic). All samples were stored at − 80 °C for maximum of 6 months before pre-analytic handling.

### Protein immunoblot and ELISA

Equal amount of extracted proteins (4.4–8.7 µg/well) was separated by 4–12% gradient Bis–Tris gels (WG1401, Invitrogen, Carlsbad, CA, USA) under denaturized conditions in ice cold MES-SDS running buffer (NP000202, Invitrogen). After gel electrophoresis, proteins were transferred onto PVDF-membranes (162-0177, Bio-Rad, Hercules, CA, USA) over 90 min, before being blocked in a 5% fat-free skimmed milk and TBS-t solution (TBS, 170-6435, Bio-Rad; 0.1% Tween-20, P5927, Sigma-Aldrich, St. Louis, MO, USA; Skim milk, 1.15363, Merck, Darmstadt, Germany) overnight at 4 °C with gentle agitation. To check and ensure successful transfer to the PVDF-membranes, all gels were incubated in Coomassie (Simply Blue SafeStain, LC6060, Invitrogen) overnight with gentle agitation at room temperature before washed in dH20 and analyzed. Blocked membranes were incubated with antibodies against HSP70 (SPA-810, Stressgen, Victoria, BC, USA) or αB-crystallin (SPA-222, Stressgen) 2 h at room temperature, followed by incubation in appropriate secondary antibody (31430, Thermo Scientific, Rockford, IL, USA) for 1 h at room temperature with gentle agitation. All antibodies were diluted in a 1% fat-free skimmed milk and TBS-t solution. Between stages, membranes were washed in TBS or TBS-t. Bands were visualized using an HRP-detection system (Super Signal West Dura Extended Duration Substrate, 34076, Thermo Scientific). Chemiluminescence was measured using a CCD image sensor (Image Station 2000R, Eastman Kodak Inc., Rochester, NY, USA) and band intensities were calculated with the Carestream molecular imaging software (Carestream Health Inc., Rochester, NY, USA). HSP27 was measured with an in-house-made double-antibody sandwich ELISA using a monoclonal capture antibody against HSP27 (25 ng/well; ADI-SPA-800, Stressgen) and a polyclonal detection antibody against HSP27 (ADI-SPA-803, Stressgen). Horseradish peroxidase conjugate was used as a secondary antibody (RPN 4301, Amersham Biosciences, GE Healthcare Life Sciences, Buckinghamshire, UK). The HSP27 assay was performed in high-binding polystyrene microplates (3590, Costar, Inc., Corning, NY, USA) using tetramethylbenzidine (TMB Solution, CL07, Calbiochem, EMD Biosciences, Germany) as substrate and 2 N sulfur acid as a stop solution. All wells were blocked for 2 h in a blocking solution (0.05% BSA, 1% BSA, 0.05% Proclin 300, pH 7.4) to prevent unspecific binding before being washed and the samples were added. Recombinant HSP27 (SPP-715, Stressgen) was used as standards (0.78–25 ng/mL). All samples were analyzed in triplicates (CV < 10%). The amount of HSP27 was determined using a filter photometer measuring optical density at 450 nm. The performance of the ELISA analyses has previously been reported (Paulsen et al. 2007).

### Statistics

All values are presented as mean ± standard deviations.

Differences in baseline characteristics were tested using a one-way ANOVA with Holm-Sidak post hoc test for multiple comparisons. Two-way ANOVA with Holm-Sidak post hoc test for multiple comparisons was used to investigate differences in muscle strength pre and post training. Student´s *t*-test was used to investigate differences between pre and post training intervention and differences between young and elderly in HSP content. A one-way ANOVA with Holm-Sidak post hoc test was applied to investigate differences between groups at post, and differences between the different age cluster analyses. To investigate numeric relationships between variables, a Pearson product-moment correlation coefficient was used. The level of significance was set to *P* ≤ 0.05. Graphpad Prism 6 (GraphPad Software Inc., La Jolla, CA, USA) was used for statistical analyses.

## Results

At baseline, the elderly groups were not different regarding age, height, body mass or BMI. (Table 1). The young participants were taller (*P* < 0.001) and stronger (higher 1RM; *P* < 0.001) than the elderly, but had the same body mass and BMI (Table 1). Over the training intervention STG, FTG and YSTG increased body mass and lean mass (Rønnestad et al., 2007; Solberg et al. 2013). We were not able to detect any content of αB-crystallin, HSP27 or HSP70 in the nuclear or cytoskeletal fraction in the biopsies from the elderly. Additionally, we were not able to detect HSP27 in the membrane fraction in the elderly.

### Muscle strength

**Fig. 1:**
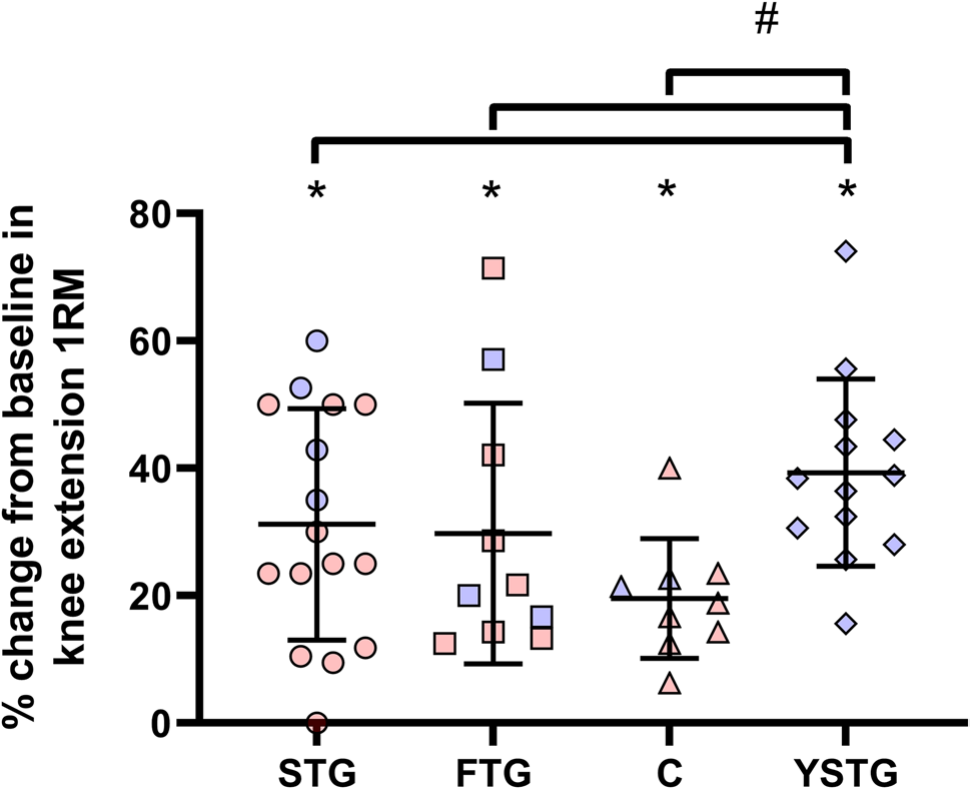
Percentage change in knee extension 1 repetition maximum (1RM). The figure displays individual values, as well as mean and standard deviations. *STG* elderly strength training group. *FTG* elderly functional strength training group. *C* elderly control group. *YSTG* young strength training group. Red indicates female participants. Blue indicates male participants. *Different from pre (*p* < 0.05). #Different between groups (*p* < 0.05)

All groups increased 1RM in knee extension after the training intervention (*P* < 0.05; Fig. 1). Between the groups of elderly, there were no group differences. However, YSTG increased more (*P* < 0.001) in muscle strength than the elderly participants after the training intervention.

### Age differences in HSP

**Fig. 2:**
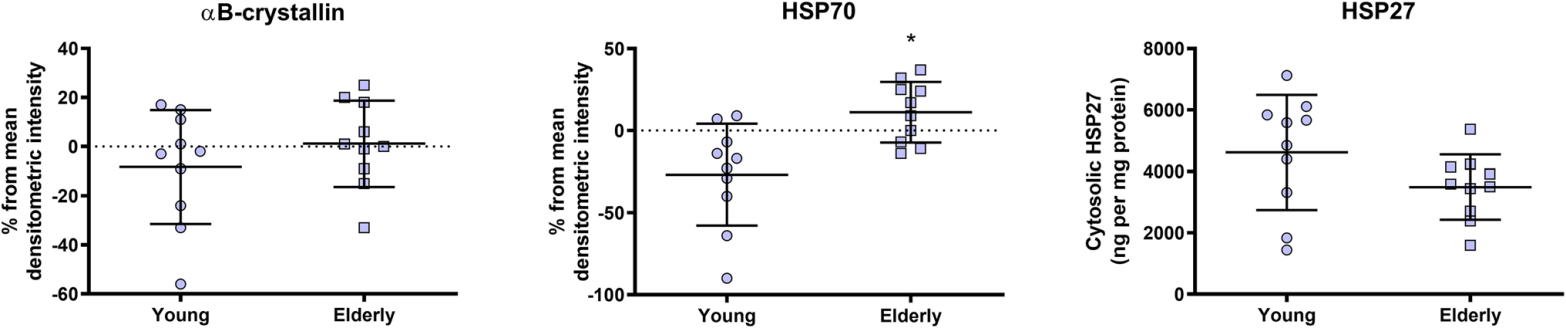
Differences in relative content of cytosolic αB-crystallin, HSP70 and HSP27 between young and elderly males based on the mean densitometric intensity. Figures display individual values, as well as mean and standard deviations. Blue indicate male participants. *Different between groups (*p* < 0.05)

To investigate any differences in baseline HSP content between young and elderly, we performed a sub-analysis of all 10 (*n* = 10) elderly men and 10 (*n* = 10) randomly selected young participants (from a total of 13). Since the group with young participants only consisted of males, we choose to only compare this group with all 10 elderly males included in the study. The analyses of αB-crystallin and HSP70 were based on an analysis of the samples within each PVDF-membrane, where all samples were analyzed relative to the mean densitometric intensity on each protein band on the PVDF-membrane. Every PVDF-membrane contained samples from both young and elderly. At baseline, elderly males had higher cytosolic HSP70 content compared to young males (*P* = 0.004; Fig. 2), whereas no age differences were observed for αB-crystallin or HSP27 (Fig. 2).

### Effects of training on HSP

**Fig. 3:**
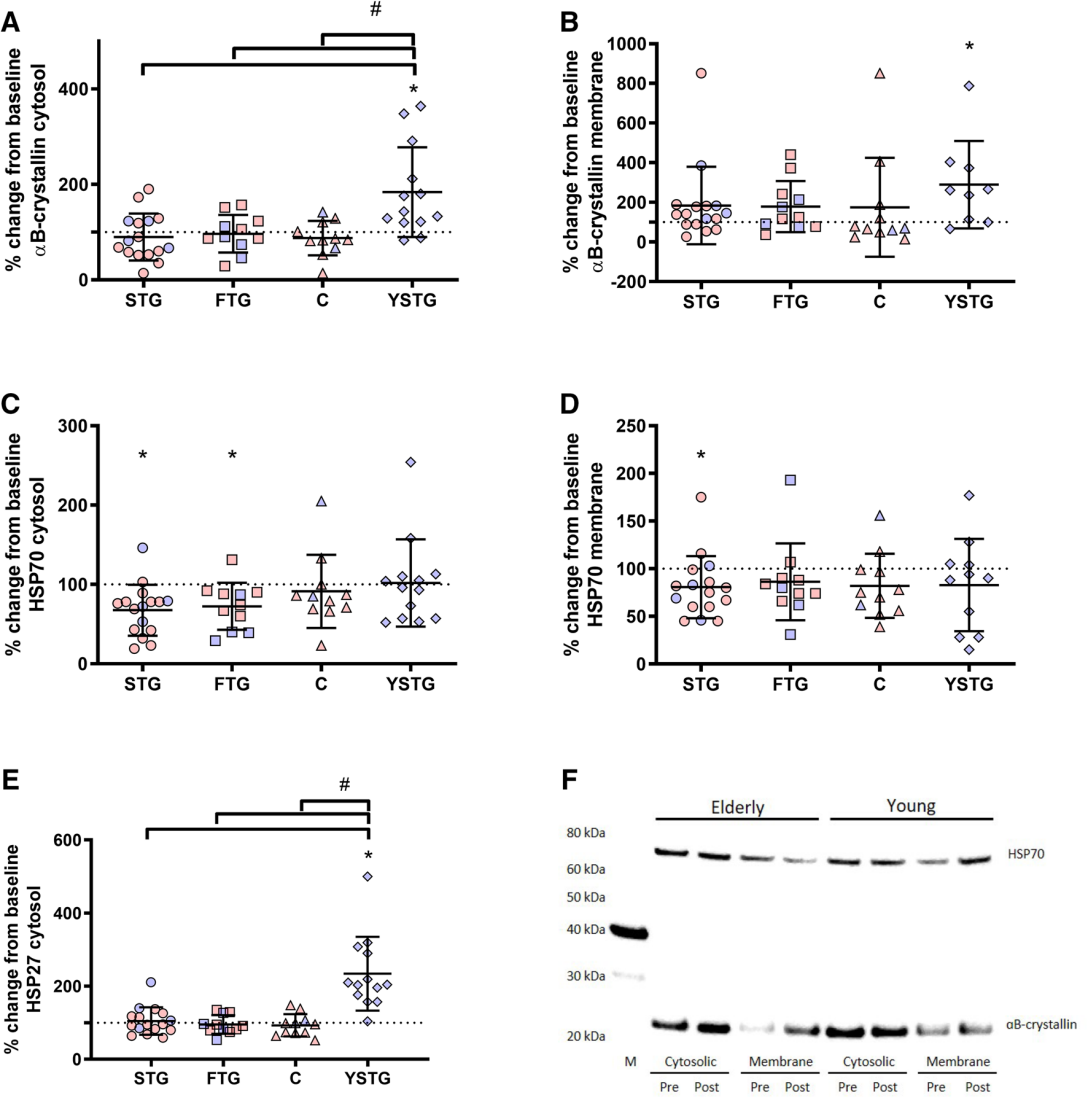
Percentage changes in cytosolic αB-crystallin (**a**), membrane bound αB-crystallin (**b**), cytosolic HSP70 (**c**), membrane bound HSP70 (**d**) and cytosolic HSP27 (**e**) after the training intervention. Representative blots are presented in F. Stippled line indicates baseline (100%). Figures display individual values, as well as mean and standard deviations. *STG* elderly strength training group. *FTG* elderly functional strength training group. *C* elderly control group. *YSTG* young strength training group. Red indicates female participants. Blue indicates male participants. *Different from pre (*p* < 0.05). #Different between groups (*p* < 0.05)

αB-crystallin did not change during the training period in the cytosolic nor membrane fraction (Figs. 3a, b) for any of the elderly training groups (STG, FTG, C). For the young participants, strength training increased αB-crystallin by 84 ± 94% (*P* = 0.008) in the cytosolic fraction and by 189 ± 221% (*P* = 0.033) in the membrane fraction (Fig. 3b). HSP70 decreased by 33 ± 32% (*P* = 0.001; Fig. 3c) in the cytosolic fraction in STG and 28 ± 30% (*P* = 0.012; Fig. 1c) in the FTG after the training intervention. However, this decrease was not significantly different from C or YSTG. No significant changes were observed in C or YSTG (Fig. 1c). In the membrane fraction, only STG decreased HSP70 by 19 ± 33% (*P* = 0.032; Fig. 3d) after the training intervention, with no changes observed in the other groups. However, this decrease was not significantly different from C or YSTG. For HSP27, no changes were observed in the elderly (Fig. 3e), whereas in the young participants (YSTG), HSP27 increased by 134 ± 1% (*P* < 0.001) after the training period (Fig. 3d), which were significantly different from all other groups (*P* < 0.001; Fig. 3e).

**Fig. 4:**
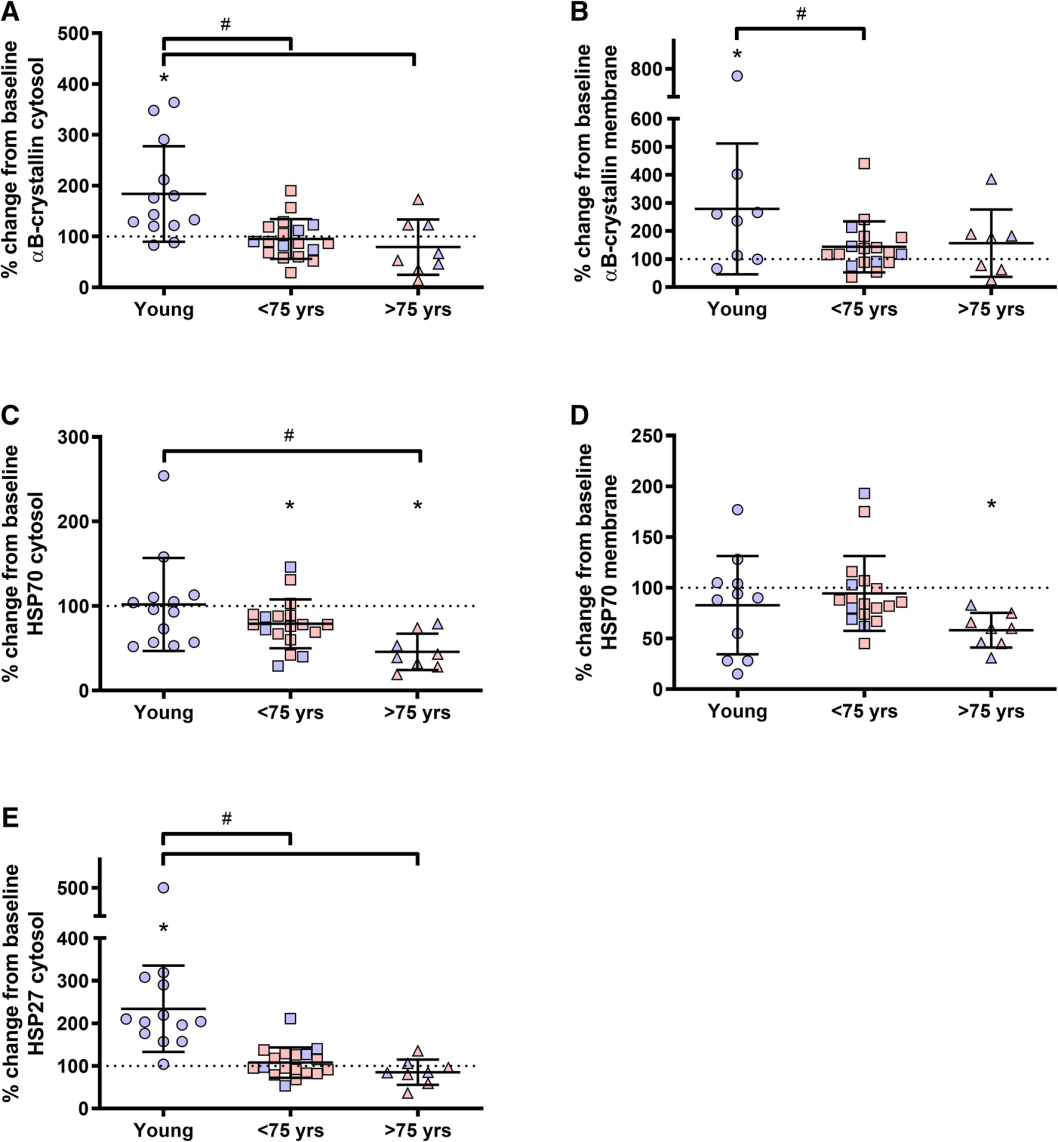
Percentage changes in cytosolic αB-crystallin (**a**), membrane bound αB-crystallin (**b**), cytosolic HSP70 (**c**), membrane bound HSP70 (**d**) and cytosolic HSP27 (**e**) after the training intervention for three different age groups performing strength training (STG and FTG combined). Stippled line indicates baseline (100%). Figures displays individual values, as well as mean and standard deviations. Red indicates female participants. Blue indicates male participants. *Different from pre (*p* < 0.05). #Different between groups (*p* < 0.05)

To further analyze the effect of age on the HSP response to resistance training, we combined STG and FTG, and subdivided the group into two groups based on age: < 75 years (*n* = 18; 72 ± 2 years) and > 75 years (*n* = 8; 82 ± 5 years). The increase in αB-crystallin observed in young were significant different from both the < 75 years and > 75 years age groups (both *P* = 0.002; Fig. 4a). In the membrane fraction, the increase observed in the young was not significantly different from the other age groups (Fig. 4b).

The sub-analyses revealed a significant decrease in cytosolic HSP70 levels post-training for < 75 years (− 21 ± 29, *p* = 0.007) and the > 75 years (− 54 ± 22%; *P* < 0.001; Fig. 4c). However, the decrease in > 75 years was only significantly different from the young (*p* = 0.008). In the membrane fraction, only > 75 years decreased HSP70 content after the training intervention (− 42 ± 17%; *P* < 0.001; Fig. 4d). However, this was not different from the other age groups. After the training period, the young increased cytosolic HSP27, which were significantly different from both the < 75 and > 75 years age groups (both *P* < 0.001; Fig. 4e).

### Correlations

Baseline HSP27 content was negatively correlated with baseline muscle strength (1RM in knee extension; *r* = − 0.469; *P* = 0.001) for all participants, and for the elderly only (*r* = − 0.357; *P* = 0.041). Independent of training group, the percentage change in 1RM over the training period correlated with baseline HSP27 content (*r* = − 0.365; *P* = 0.013).

No correlations were found between percentage change in muscle strength and change in HSP70 or αB-crystallin in the cytosolic or membrane fraction.

## Discussion

To our knowledge, this is the first study investigating changes in HSP levels in response to different strength training modalities in skeletal muscles from elderly participants. The main findings were that basal HSP70 levels were higher in elderly than in young, and that regular strength training over 12 weeks reduced HSP70 levels in the elderly. Furthermore, the decline in HSP70 with training was more pronounced in the oldest lending support to the theory that regular strength training can normalize some of the increases in cellular stress associated with aging. Finally, strength training increased levels of small HSPs in young, but not in the elderly.

### HSP70

The higher muscular HSP70 levels at baseline in the elderly compared to the young is in line with previous studies in humans (Joseph et al. 2012; Thalacker-Mercer et al. 2010; Cobley et al. 2014; Aas et al. 2019). High expression of HSP70 can, as suggested in different aging models, indicate an unbalanced and stressful environment in the cell caused by aging processes (for review see Tower 2009). The aging process is complex, and has several potential mechanisms and explanations (López-Otín et al. 2013). As stated, low-grade inflammation alters muscle cell homeostasis and has been shown to increase HSP70 content in the elderly, and the increased low-grade inflammation has been linked to increased oxidative stress (Meng and Yu 2010). Increased oxidative stress influences the oxidative status in cells and accumulates damage over time which increases levels of the HSPs (Bond et al. 2018; Zou et al. 1998). Herein, we report that the initial high levels of HSP70 in the elderly were reduced by both strength-training interventions. This is in line with our observations of reduced HSP70 levels after strength training in elderly (66 years old) male prostate cancer patients undergoing androgen deprivation therapy (Nilsen et al. 2016), the finding of Beltran Valls et al. (2013), reporting reduced HSP levels in old participants after 12 weeks resistance exercise, and the observation of reduced HSP70 levels in diabetic rat muscles undergoing resistance training (Molanouri Shamsi et al. 2016). Since HSPs tend to translocate in response to acute stress, there is the possibility that the decreased cytosolic HSP70 observed in the elderly were related to translocation to other cell compartments. However, in this study, we also analyzed the membrane fraction, and the nuclear and cytoskeletal (not reported) where we could not find any HSPs (under the detection limits). Importantly, we did not observe any increase in HSP70 in the membrane fraction, nor the other fractions. Consequently, translocation to other compartments could not explain the reduction in HSP70 in the cytosolic fraction. In fact, HSP70 was reduced in the membrane fraction in the STG group indicating a reduced total content of HSP70. Based on the assumption that initial high HSP70 in the elderly indicates a general increase in cellular stress with aging, we argue that strength training may normalize muscle cell stress, e.g., from oxidative damage. Our study also show that this normalization is independent of the work performed during the training intervention since it occurs in both the STG and FTG. This suggests that reduction in HSP70 is independent of training volume and exercise intensity, at least with in the variations investigated in this study.

Training studies in elderly report increased enzymes with antioxidant properties, e.g., SOD2 and peroxiredoxin (Gliemann et al. 2013; Cobley et al. 2014) and thus improve the oxidative status in aging muscle cells. Simar et al. (2007) reported lower levels of HSP72 (HSP70) in leukocytes of elderly with the highest physical activity level compared to elderly with medium to low level of physical activity. Whether this also applies to other tissues unknown, but the observation of reduced muscular HSP70 levels with strength training in this study supports the idea of a general positive effect of regular exercise on cellular homeostasis. We must emphasize that our analyses on baseline HSP70 were only done for males. However, the decrease was seen in both males and females, which makes it plausible that both elderly males and females had high HSP70 levels at baseline. With the possible combination of ineffective autophagy processes in aged (Zhou et al. 2017), damaged and non-functional proteins can accumulate, and HSP levels can increase as a compensating mechanism (Aas et al. 2019). Consequently, it might be the combination of disuse and aging that exerts the negative impact on aging skeletal muscle, and regular resistance exercise can, at least to some extent, reverse some of these changes associated with aging.

### Small HSPs

The present study is in line with our previous study showing that healthy elderly and young have the same basal levels of αB-crystallin (Aas et al. 2019). αB-crystallin levels are seemingly increasing with reduced functional capacity and can be viewed as a hallmark of frailty in elderly (Aas et al. 2019). Since our participants did not show any frailty, this could explain why our elderly had similar αB-crystallin as what was seen in muscles from young.

In response to training, we observed no changes in αB-crystallin nor HSP27 in the elderly after any of the strength training modalities (STG and FTG). Previous training studies are contradictory, with studies reporting increased αB-crystallin and HSP27 in untrained young participants after strength training (Gjøvaag and Dahl 2006; Paulsen et al. 2011), and unchanged in young untrained females (Cumming et al. 2017). In muscles from old rodents, it has been reported depressed protein expression of HSP25 compared to young acutely after an exercise session and after a period of strength training (Murlasits et al. 2006; Vasilaki et al. 2006), and this could relate to impaired activation and content of HSF-1 (Locke and Tanguay 1996). This would mean that the levels of αB-crystallin and HSP27 in the exercised muscles are either sufficient concerning the exercise strain or it does not keep up the pace with the increased loads and strain put on the muscles from resistance training. Furthermore, the elderly in the present study showed higher levels of HSP70. A possibility is that the high levels of HSP70 might have compensating roles in the cell. Thus, there is a possibility that there is no need for more αB-crystallin and HSP27 at this stage of aging or for training elderly.

## Conclusion

The aim of the present study was to investigate the training response on HSP content in elderly participants in response to two types of strength training modalities.

Herein, we demonstrate that strength training decreases and stabilizes HSP70 levels in the elderly with initially high levels, whereas no changes were seen in the young training group or elderly control group. Furthermore, only the young participants increased the content of the small HSPs αB-crystallin and HSP27. Overall, this indicates a different response to training between the elderly and young participants, and we suggest that the reduced HSP70 levels observed with training in the elderly indicate a normalization of the general cellular stress. In respect to the HSP response to exercise training, we found no differences between heavy load strength training and functional strength training in the elderly and no differences between elderly male and females.

## Supplementary Information

Below is the link to the electronic supplementary material.

Supplementary file1 (DOCX 14 KB)

## Abbreviations

ANOVA: Analysis of variance
BMI: Body mass index
HSF-1: Heat shock factor 1
HSP: Heat shock protein
HSP25: Heat shock protein 25
HSP27: Heat shock protein 27
HSP70: Heat shock protein 70
HSP72: Heat shock protein 72
RM: Repetition maximum
SOD2: Superoxide dismutase 2
