# Supplementary material for: Muscular HSP70 content is higher in elderly compared to young, but is normalized after 12 weeks of strength training
Source: Eur J Appl Physiol. 2021 Mar 7;121(6):1689–99. doi: 10.1007/s00421-021-04633-4 (PMC8144120; doi:10.1007/s00421-021-04633-4)
Supplement: Supplementary file 1 — Supplementary file1 (DOCX 14 KB) [file 421_2021_4633_MOESM1_ESM.docx]

Supplemental data

Table s1. Training program for STG.

| **Week** | **Body part** | **No. of exercises** | **Day 1**  **(series x intensity)** | **Day 2**  **(series x intensity)** | **Day 3**  **(series x intensity)** |
| --- | --- | --- | --- | --- | --- |
| 1-4 | Upper body | 3 | 1x 12 RM | 1x 80% of 10 RM | 1x 8 RM |
|  | Lower body | 5 | 2x 12 RM | 2x 80% of 10 RM | 2x 8 RM |
| 5-6 | Upper body | 3 | 1x 10 RM | 1x 80% of 10 RM | 1x 6 RM |
|  | Lower body | 5 | 2x 10 RM | 2x 80% of 10 RM | 2x 6 RM |
| 7-8 | Upper body | 3 | 2x 10 RM | 2x 80% of 10 RM | 2x 6 RM |
|  | Lower body | 5 | 3x 10 RM | 3x 80% of 10 RM | 3x 6 RM |
| 9-12 | Upper body | 3 | 2x 8 RM | 2x 80% of 10 RM | 2x 4 RM |
|  | Lower body | 5 | 3x 8 RM | 2x 80% of 10 RM | 3x 4 RM |

RM, repetition maximum.

Table s2. Training program for FTG.

| **Week** | **No. of exercises**  **(upper-/lower body)** | **Exercise set** | **Day 1**  **(series x rep)** | **Intensity** | **Day 2 (series x rep)** | **Intensity** | **Day 3 (series x rep)** | **Intensity** |
| --- | --- | --- | --- | --- | --- | --- | --- | --- |
| 1-6 | 7 (4/3) | 1 | 1x 15 | 20 RM | 1x 10 | 20 RM | 1x 15 | 20 RM |
|  |  | 2 | 1x 15 | 15 RM | 1x 10 | 15 RM | 1x 15 | 15 RM |
| 6-12 | 8 (4/4) | 1 & 2 | 2x 12 | 12 RM | 2x 10 | 15 RM | 2x 12 | 12 RM |

Rep, repetitions. RM, repetition maximum.

Table s3. Training program for YSTG.

| **Week** | **No. of exercises**  **(upper-/lower body)** | **No. of training sessions per week** | **Training load**  **(series x intensity)** |
| --- | --- | --- | --- |
| 1-2 | 8 (5/3) | 3 | 3x 10 RM |
| 3-4 |  |  | 3x 8 RM |
| 5-11 |  |  | 3x 7 RM |

RM, repetition maximum.
